# Evaluating the features of breast lesions identified by bimodal breast examination: a real-world study

**DOI:** 10.3389/fonc.2024.1406144

**Published:** 2024-07-26

**Authors:** Xiaoxi Huang, Shuai Zhao, Wanqian Chen, Bin Sun, Zhenchuan Lin, Haomin Yang

**Affiliations:** ^1^ Department of Breast, Fujian Maternity and Child Health Hospital, College of Clinical Medicine for Obstetrics & Gynecology and Pediatrics, Fujian Medical University, Fuzhou, Fujian, China; ^2^ Computer Technology Department, Fujian Obstetrics and Gynecology Hospital, Fuzhou, Fujian, China; ^3^ Division of Birth Cohort Study, Fujian Maternity and Child Health Hospital, Affiliated Hospital of Fujian Medical University, Fuzhou, Fujian, China; ^4^ Fuzhou Bochuang Medical Laboratory Co., Ltd., Fuzhou, Fujian, China; ^5^ Department of Epidemiology and Health Statistics, School of Public Health, Fujian Medical University, Fuzhou, Fujian, China

**Keywords:** breast lesion, breast cancer, palpation imaging, ultrasound, mammogram

## Abstract

**Background:**

Several image-based diagnostic methods have been developed to examine the features of breast lesions among women, while the value of combining palpation imaging and ultrasound by a bimodal breast examination system is still unknown.

**Methods:**

A real-world study was conducted among 424 patients who visited Fujian Maternal and Child Health Hospital and Fujian Obstetrics and Gynecology Hospital, and used the Bimodal Breast Exam (BBE) systems which combines palpation imaging and ultrasound imaging. Among them, 97 patients had additional ultrasound, mammogram, or pathological examination. These patients were used to evaluate the consistency and efficacy of the BBE in interpreting the features of breast lesions as compared to results of ultrasound, mammogram, and pathological examinations.

**Results:**

The BBE system detected 1517 lesions with palpation imaging, 1126 lesions with ultrasound examination (950 solid lesions and 176 cysts), and 391 non mass lesions. Among them, 404 patients were diagnosed as benign and 20 were diagnosed as malignant tumor. However, 12, 9 and 4 cases were diagnosed as malignant tumors by ultrasound, mammogram and pathological examination, respectively. Compared with the integrative results of ultrasound, mammogram and pathology, the sensitivity of BBE is 55.6%, and the specificity is 90.9%, with a kappa coefficient of 0.387 (0.110, 0.665), indicating moderate consistency.

**Conclusions:**

In clinical practice, BBE can be used to evaluate features of breast lesions with a high specificity. The diagnostic efficacy is comparable to the integrative results of ultrasound, mammography, and pathological examination.

## Introduction

At present, the main diagnostic methods to determine the feature of breast lesions include clinical breast examination (CBE), breast palpation imaging (BPI), ultrasound (Breast Ultrasound System, BUS), mammography (MAM), and magnetic resonance imaging (MRI) (1). Due to China’s large population base and uneven distribution of medical resources, breast cancer screening is conducted in a centralized manner, and China needs to find efficient, safe and convenient approaches for breast cancer screening at this stage.

The Bimodal Breast Examination (BBE) system integrates palpation imaging and ultrasound imaging, and is performed by a single examiner using a sequential examination method of palpation followed by ultrasound. The palpation imaging system can simulate the process of doctor’s manual palpation, sensitively detect abnormalities in tissue elasticity, and instantly output structured data reflecting pathological characteristics of lesions, such as lesion hardness, size, shape, activity, surface smoothness, internal homogeneity, etc., achieving standard visualization of CBE (2). Ultrasound then selectively examines the cystic and solid properties of the lesion and its relationship with surrounding tissues, and these two mutually confirm each other, which can improve the efficiency and accuracy of the examination.

Compared with traditional breast examination methods, BBE’s palpation imaging achieves standard visualization of CBE, eliminating the influence of individual differences and subjective descriptions from the doctors. BBE’s palpation imaging further reduces the excessive reliance of ultrasound imaging on the examiner’s expertise and the time spent for searching the lesion using ultrasound (3). Despite these, efficacy of BBE has not been evaluated. Therefore, this study aims to explore the efficacy of BBE in distinguishing breast lesions as compared to the main diagnostic methods used at present.

## Materials and methods

### Study population

We conducted a real-world study on clinical data of patients who took BBE in the Department of Breast, Fujian Maternity and Child Health Hospital, and Fujian Maternal and Obstetrics and Gynecology Hospital from July to December 2023. Patients with a diagnosis of breast cancer, presence of prosthesis implantation, or a history of breast surgery were excluded. Finally, a total of 424 patients with age 15–65 years old were included. The patients were linked to electronic medical records by patient ID to obtain information on ultrasound, mammography, and pathological data. Among them, 71 ultrasound records, 23 mammogram records, and 41 pathological records were obtained, corresponding to 97 patients. The study was approved by the ethics committee in Fujian Maternity and Child Health Hospital (2023KY048).

### Bimodal breast examination system

In the breast examination room, patients were instructed to lie on their backs, expose both breasts, and use a bimodal breast examination system (Fuzhou Yijiajian Technology Co., Ltd., BE5W) for examination. Before starting the examination, symptoms and signs such as breast pain and nipple discharge were obtained through consultation and visual examination. During the examination process, the system first used a palpation probe perpendicular to the surface of the breast for routine clockwise pressure scanning. The probe is made up of hundreds of pressure sensors, and the probe area pressed on the breast is about 35×45mm. The force applied is in the range of 0–2kg, and the obtainable lesion size is 5mm. Palpation imaging is a tissue elastography examination technique that utilizes the stress-strain principle of physics to achieve tissue elastography (1). The elastometric method herein applied is a branch of elastography, which differs from ultrasonic and magnetic resonance elastography in that it evaluates soft tissue mechanical structure using stress data rather than dynamic or static strain data. Under the same stress, if the elastic parameter (commonly understood as hardness) of a certain or partial tissue within a soft tissue organ is high, the strain caused is relatively small. Conversely, if the elastic parameter is low, the corresponding strain is relatively large.

When simulating hand palpation for tactile pressure examination, palpation imaging detects lesions based on the difference in hardness between the local tissue of the lesion and the surrounding normal breast tissues. As long as there is a difference in hardness, it may be sensitively detected and the coronal image of the lesion can be displayed in the form of pressure distribution in real-time. Palpation imaging also eliminates the dependence of clinical manual palpation on the individual experience of the examiner, as well as the limitations of objective recording of examination information. The occurrence of various benign and malignant lesions in soft tissue organs can lead to varying degrees of increase in local hardness, especially in malignant breast solid tumors. After finding the lesion, it adjusted the pressure to focus on scanning and recording until the image of the lesion was complete and stable. Then it stopped the examination, and marked the location of the lesion on the breast diagram.

Subsequently, using an ultrasound linear array probe (7MHz) in the same position, the BBE system performed examinations in both anterograde and perpendicular directions to the mammary duct at the location where the lesion is palpated. When the ultrasound image was clear and stable, it measured and annotated the extracted images, described the examination findings, and saved them. In the final step, it outputted structured data such as the location, hardness, size, shape, aspect ratio, boundary smoothness, surface smoothness, internal homogeneity, and blood flow of the lesion. Doctors then used these structured data to classify the lesions, based on the Breast Imaging Reporting and Data System (BI-RADS). BI-RADS 4 and above were classified as suspected malignancy (positive), and BI-RADS 3 and below were suspected benign (negative).

### Breast ultrasound, mammogram and pathological examination

High-frequency linear array probes of ultrasound equipment such as ResonaR9Q, VOLUSON E10, LOGIQ S8, and LOGIQ fortis were used for ultrasound examinations of the breast and superficial lymph nodes according to standards. Digital mammography machines were used to perform mammograms. The integrative results for the 97 patients with breast ultrasound, mammogram or pathological examination were judged according to the evidence level: pathological results>mammogram results>ultrasound results if they had more than two of these examinations. Ultrasound and mammograms were both judged according to the BI-RADS.

### Statistical analysis

The categorical variables are represented by n (%),and continuous variables are represented by mean ± standard deviation. We further used kappa analysis to evaluate the consistency between BBE and the integrative judgment of pathology, mammogram, and ultrasound examinations in R 4.2. *P*<0.05 indicates a statistically significant result. Non-inferiority test was used to evaluate the non-inferiority of BBE as compared to the integrative results, and the Tango asymptotic score was used to estimate confidence interval by R package ‘ratesci’ (4).

## Results

### BBE detected lesions and lesion types

**Figure 1:**
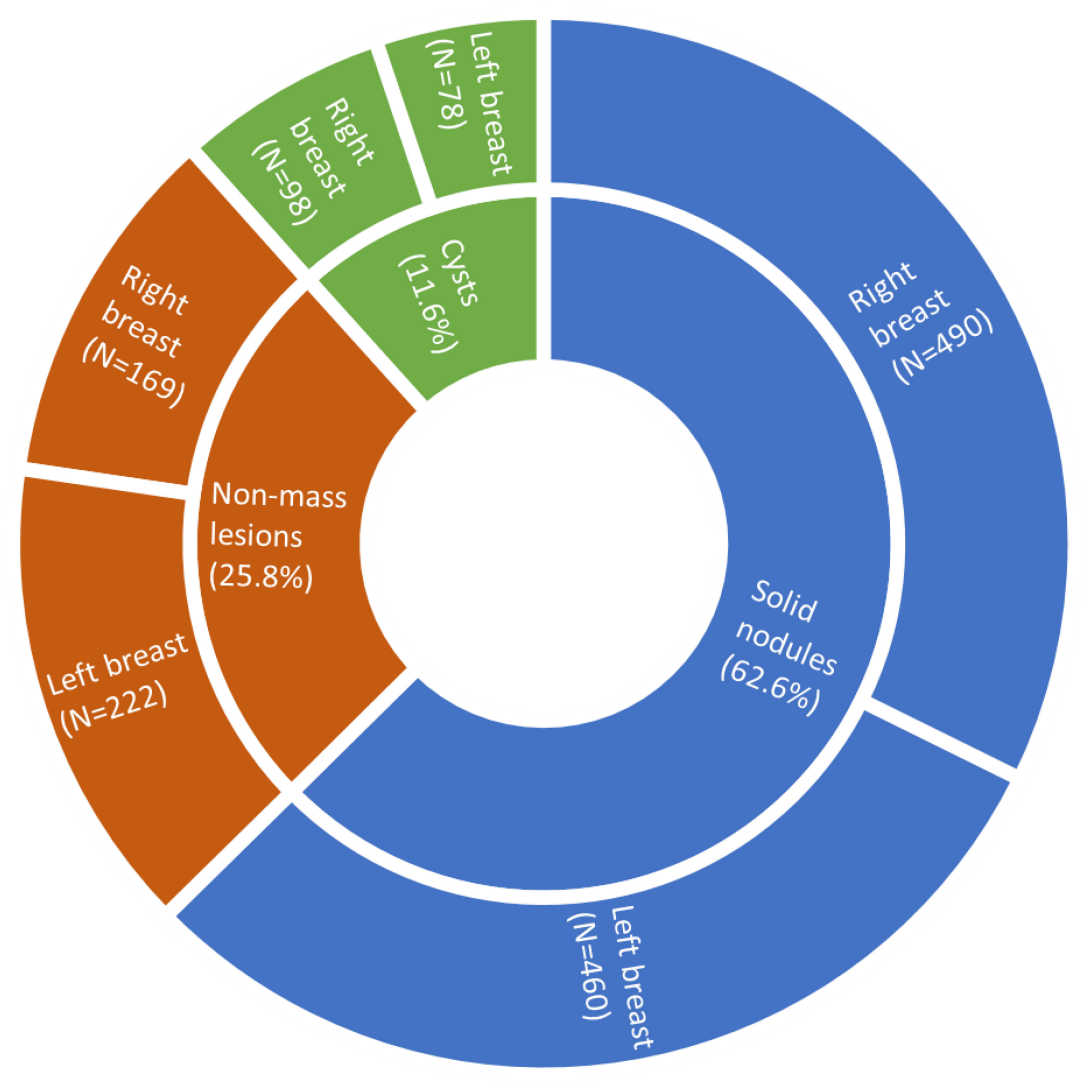
The breast lesions and lesion types detected by BBE.

**Figure 2:**
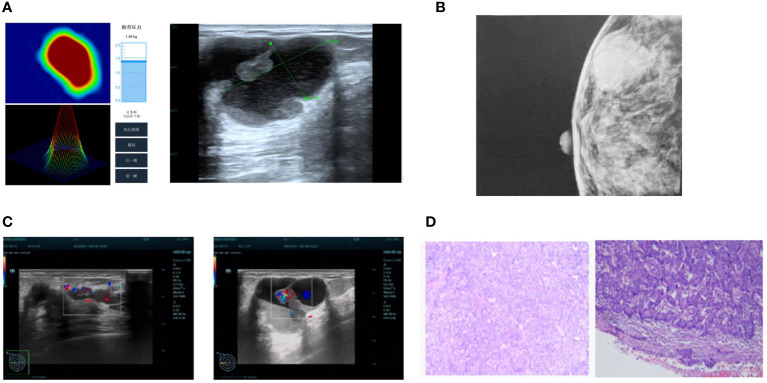
The visual difference between different methods for one patient. **(A)** Bimodal Breast Examination; **(B)** Mammography; **(C)** Ultrasound; **(D)** Pathology.

The mean age of the 424 patients was 32.4 ± 6.9 years old. BBE’s palpation imaging detected a total of 1517 lesions, among which 1126 lesions were also detected by ultrasound examination. In all these lesions (**Figure 1**), 950 (62.6%) were solid nodules, 176 (11.6%) were cysts and 391 were non-mass lesions (25.8%). The BBE system classified 404 cases as benign and 20 cases as malignant. An example of the images by different detection methods for a patient is shown in **Figure 2**.

### Comparison between BBE and integrative results from pathology, mammogram and ultrasound

**Figure 3:**
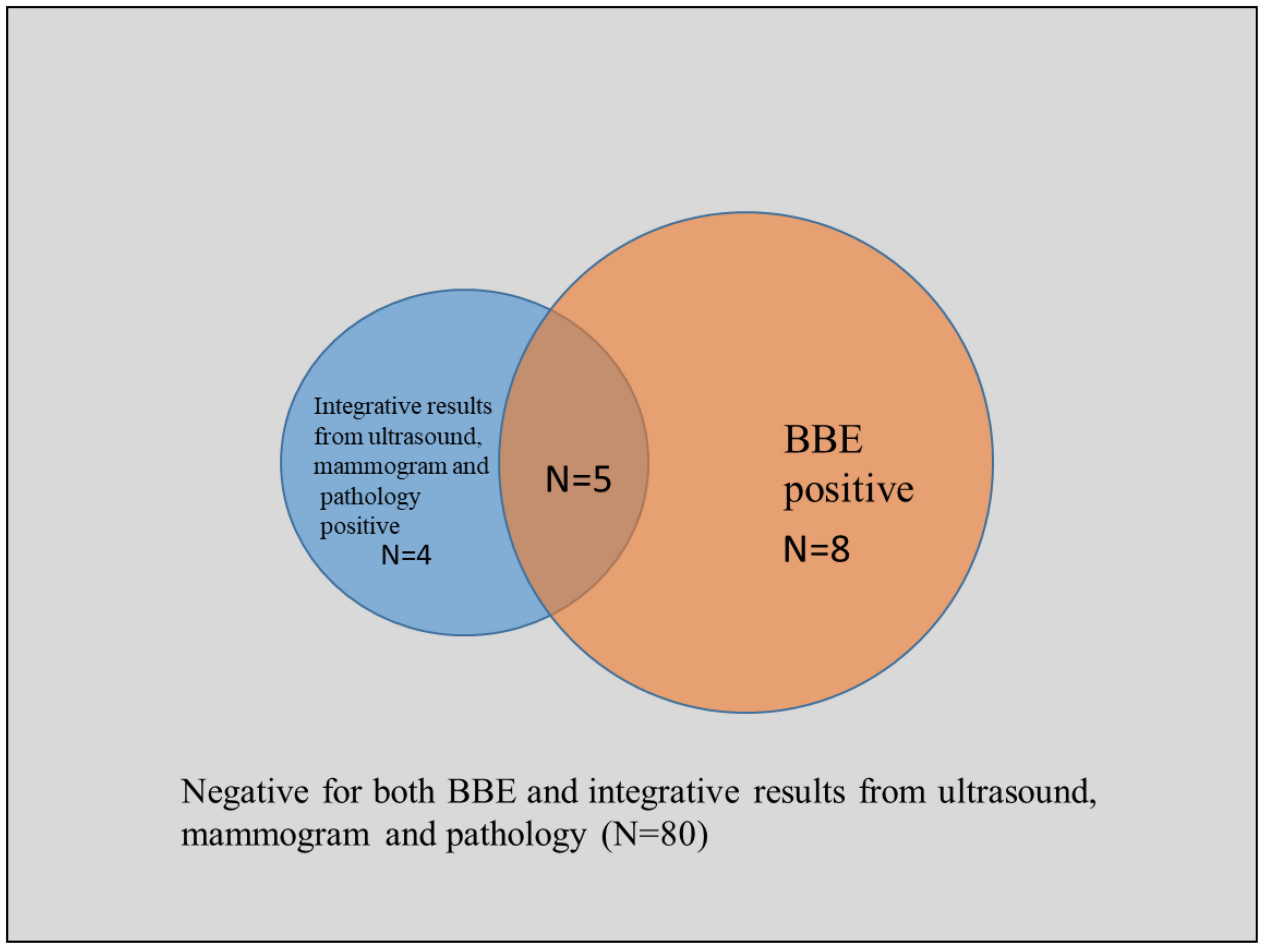
Venn plot of the detection results from BBE and integrative results from ultrasound, mammogram and pathology.

Among the 97 patients with pathology, mammogram or ultrasound data, 9 patients were considered as positive and 88 were negative, while 13 were considered as positive using BBE system (**Figure 3**).

Compared with the integrative results of pathology, mammogram and ultrasound examination, the sensitivity of BBE examination is 55.6%, the specificity is 90.9%, the positive predictive value is 38.5%, the negative predictive value is 95.2%, and the diagnostic accuracy is 87.6%. The consistency analysis showed a kappa coefficient of 0.387 (0.110, 0.665), indicating moderate consistency.

The non-inferiority comparison between BBE and integrative results of pathology, mammogram and ultrasound examination showed that BEE examination is not inferior to the integrative results. The 95% confidence interval for the rate difference in detecting positive results between the two is (-0.033, 0.121), and the confidence interval is located to the right of the non-inferiority limit=-0.10, indicating the validity of the non-inferiority conclusion.

## Discussion

The BBE system is a new type of breast examination method that integrates palpation imaging and ultrasound imaging. The palpation imaging has been clinically recognized for its effectiveness in detecting and diagnosing breast lesions, such as the structured data and representative features of lesion hardness, size, shape, and surface smoothness output by breast palpation imaging (2, 5), which have statistical significance to distinguish benign and malignant breast lesions (6, 7).

The results of this study suggested that the palpation imaging of BBE detected more positive lesions than ultrasound. This finding is consistent with previous study showing that greyscale ultrasound images and shear wave ultrasound elastography are extremely sensitive for detection of breast malignancy (8). As the proportion of solid lesions confirmed by ultrasound after palpation imaging is about 82.6%, it is speculated that the main reason for the more lesion detected is that palpation imaging detects more lesions based on the difference in tissue elasticity between the lesion and surrounding normal breast tissue. When palpation imaging finds a lesion, it can prompt ultrasound to carry out corresponding focused examinations, such as adding color Doppler to display rich blood flow signals in non-mass lesions, thereby reducing the probability of missed diagnosis of non-mass breast cancer (9).

In case of the fact that non-mass breast cancer mostly presents diffuse and regional structural disorder, duct expansion, and structural distortion, and often has no clear boundary and space occupying effect, ultrasound has low efficiency for non-mass breast cancer detection, which has become a difficult issue in ultrasonic clinical examination (10). Despite this, palpation imaging can reduce the time required for ultrasound scanning of all parts of the breast, thereby improving the efficiency of the examination. For example, in this study, the average time for 424 cases of BBE examination was 4.87 minutes, while traditional breast palpation combined with ultrasound examination took more than 15 minutes.

## Conclusions

In the real-world scenario, BBE system can perform two types of examinations: palpation and ultrasound. It can effectively complete breast examinations while improving examination efficiency, with a moderate consistency as compared to the integrative results from ultrasound, mammogram, and pathological examination.

## Data availability statement

The raw data supporting the conclusions of this article will be made available by the authors, without undue reservation.

## Ethics statement

The studies involving humans were approved by Fujian Maternity and Child Health Hospital. The studies were conducted in accordance with the local legislation and institutional requirements. The participants provided their written informed consent to participate in this study.

## Author contributions

XH: Conceptualization, Data curation, Formal analysis, Methodology, Software, Writing – original draft, Writing – review & editing. SZ: Data curation, Formal analysis, Investigation, Software, Writing – original draft, Writing – review & editing. WC: Formal analysis, Methodology, Software, Writing – original draft, Writing – review & editing. BS: Formal analysis, Investigation, Writing – review & editing. ZL: Resources, Writing – review & editing. HY: Writing – review & editing.

## References

[1] Wang B Fu J. Diagnostics of Breast Palpation Imaging. Beijing: Science Press (2016).

[2] Zhang X Sun S Wang H. Intelligent diagnosis model and method of palpation imaging breast cancer based on data mining. Big Data Res. (2019) 5:68–76. doi: 10.11959/j.issn.2096-0271.2019005

[3] Kaufman CS Jacobson L Bachman BA Kaufman LB. Digital documentation of the physical examination: moving the clinical breast exam to the electronic medical record. Am J Surg. (2006) 192:444–9. doi: 10.1016/j.amjsurg.2006.06.006 16978946

[4] Fagerland MW Lydersen S Laake P. Recommended tests and confidence intervals for paired binomial proportions. Stat Med. (2014) 33:2850–75. doi: 10.1002/sim.6148 24648355

[5] Song Y Zhang RZ Gao GH Zhou CW. Palpation imaging system in differentiating benign and Malignant breast lesions: Compared with physical examination, mammography and ultrasonography. Chin J Med Imaging Technol. (2014) 30:527–30.

[6] Wu J Chen W Mei Z Wang D Chai W Zhan W. et al: The value of palpation imaging in the diagnosis of breast diseases. Chin J Pract Surg. (2012) 32:390–4.

[7] Egorov V Kearney T Pollak SB Rohatgi C Sarvazyan N Airapetian S. Differentiation of benign and Malignant breast lesions by mechanical imaging. Breast Cancer Res Treat. (2009) 118:67–80. doi: 10.1007/s10549-009-0369-2 19306059 PMC2803347

[8] Evans A Whelehan P Thomson K Brauer K Jordan L Purdie C. Differentiating benign from Malignant solid breast masses: value of shear wave elastography according to lesion stiffness combined with greyscale ultrasound according to BI-RADS classification. Br J Cancer. (2012) 107:224–9. doi: 10.1038/bjc.2012.253 PMC3394981 22691969

[9] Zhang W Xiao X Xu X Liang M Wu H Ruan J. Non-mass breast lesions on ultrasound: feature exploration and multimode ultrasonic diagnosis. Ultrasound Med Biol. (2018) 44:1703–11. doi: 10.1016/j.ultrasmedbio.2018.05.005 29861297

[10] Wang Z Tang J Li J Wan W Xu J. Ultrasound diagnosis of non-mass-like breast lesions. Chin J Med Imaging. (2013) 21:13–5.

